# Diagnostic Accuracy of Cerebrospinal Fluid (CSF) Adenosine Deaminase (ADA) for Tuberculous Meningitis (TBM) in Adults: A Systematic Review and Meta-Analysis

**DOI:** 10.7759/cureus.39896

**Published:** 2023-06-03

**Authors:** Manoj Kumar Prasad, Amit Kumar, Neelam Nalini, Pramod Kumar, Brajesh Mishra, Dipti Lata, Chanchal Ashok, Dewesh Kumar, Sujeet Marandi, Divakar Kumar, Shreya Singh, Mayank Mahajan

**Affiliations:** 1 Internal Medicine, Rajendra Institute of Medical Sciences, Ranchi, IND; 2 Laboratory Medicine, Rajendra Institute of Medical Sciences, Ranchi, IND; 3 Obstetrics and Gynecology, Rajendra Institute of Medical Sciences, Ranchi, IND; 4 Biochemistry, Rajendra Institute of Medical Sciences, Ranchi, IND; 5 Pulmonary Medicine, Rajendra Institute of Medical Sciences, Ranchi, IND; 6 Zoology, Ranchi University, Ranchi, IND; 7 Pathology, Rajendra Institute of Medical Sciences, Ranchi, IND; 8 Community Medicine/Preventive and Social Medicine, Rajendra Institute of Medical Sciences, Ranchi, IND; 9 Medicine, Rajendra Institute of Medical Sciences, Ranchi, IND

**Keywords:** meta-analysis, systematic review, diagnostic accuracy, meningitis, tuberculous, csf-ada

## Abstract

Tuberculous meningitis is the most serious complication of tuberculosis. Early diagnosis is crucial to start relevant treatment to prevent death and disability. Electronic databases PubMed, Google Scholar, and Cochrane Library were used to find relevant articles from January 1980 to June 2022. The random-effect model in terms of pooled sensitivity, specificity, and diagnostic odds ratio (DOR) with 95% confidence interval was adopted to derive the diagnostic efficacy of cerebrospinal fluid (CSF) adenosine deaminase (ADA) for the diagnosis of tuberculous meningitis (TBM) in adult patients.

A total of 22 studies (20 prospective and two retrospective data) have been included in this meta-analysis, having 1927 participants. We perceived acceptable pooled sensitivity, specificity, summary receiver operating characteristics (SROCs), and diagnostic odds ratio (DOR) of 0.85 (95% CI: 0.77-0.90), 0.90 (95% CI: 0.85-0.93), 0.94 (95% CI: 0.91-0.96) and 48 (95% CI: 26-86), respectively, for CSF-ADA for differentiating TBM from non-TBM in adult patients. To ascertain the certainty of evidence for CSF-ADA as a diagnostic marker for TBM, Grading of Recommendations, Assessment, Development, and Evaluation (GRADE) analysis was used. CSF-ADA is an auspicious diagnostic test with a high degree of specificity and acceptable sensitivity for the diagnosis of tuberculous meningitis, however, with very low certainty of evidence.

## Introduction and background

Tuberculosis caused by *Mycobacterium tuberculosis* bacteria is a common infection prevalent in Southeast Asian and African countries. The most common organ affected is the lung (pulmonary TB) but other organs are also affected like lymph nodes, pleura, bone, brain, etc. (extra-pulmonary TB). All over the world, about 10 million people have been affected by tuberculosis and about 1.5 million died in 2020 due to its complications following lack of treatment, under treatment, or due to treatment failure [1]. Tuberculous meningitis (TBM) is the most serious complication of tuberculosis. Southeast Asia and Africa account for about 70% of TBM cases and 48% of death of adults due to TBM in 2019 [2].

TBM is diagnosed on the basis of clinical findings like presence of fever, headache, vomiting, nuchal rigidity, seizure, and focal neurological deficit along with various investigations like physical, chemical, cellular examination, Ziehl-Neelsen staining/culture of cerebrospinal fluid (CSF) following lumbar puncture, CT/MRI of brain or clinical response to anti-tuberculous drugs [3].

In case of TBM, CSF is straw in color, containing more lymphocytes than neutrophils, raised protein, and sugar less than the corresponding blood sugar. But the similar chemical findings may be present in other conditions like bacterial or pyogenic meningitis. In TBM, CSF smear microscopy has poor sensitivity for detecting *M. tuberculosis*. Mycobacterial culture in solid media like Lowenstein-Jensen media takes longer time (about eight weeks), however, has higher sensitivity (50-60%). Bactec MGIT 960 (Franklin Lakes, NJ: BD Biosciences) again takes longer time though lesser than the culture result (18 days vs. 38 days). The nucleic acid amplification tests (NAATs) are rapid to perform with the advantage of simultaneous detection of drug resistance but their sensitivity and specificity are lesser for non-respiratory samples. The Xpert MTB and RIF ultra (Xpert ultra) can detect *M. tuberculosis *and rifampicin resistance within 2 hours but has lower sensitivity to detect TBM except in TBM with human immunodeficiency virus infection. Moreover, due to low negative predictive value (NPV), they cannot rule out TBM. Brain imaging by CT scan and MRI can detect lesions due to TBM but these lesions are not specific and may be found in other infections or non-infectious conditions. Interferon-Gamma Release Assays (IGRA) is used to detect latent TB irrespective of HIV status. It can help to detect latent TB, however, can be positive in TB disease as well [4].

Measurement of CSF-adenosine deaminase (ADA) is a rapid, cheaper, and easily accessible, most common immunodiagnostic method, for TBM diagnosis. ADA is an enzyme released by the T-lymphocytes with cell-mediated immune response to tubercle bacilli [5]. A recent meta-analysis by Pormohammad et al. in 2017 of 20 studies on the diagnostic accuracy of CSF-ADA for TBM has reported pooled sensitivity and specificity of 89% and 91%, respectively [6]. However, this meta-analysis has included both adults as well as children as their study population. After this meta-analysis, several new studies have appeared in the literature.

Because of increased incidence of traumatic lumbar puncture in neonates and children, there can be confusing CSF examination results [7]. Moreover, worldwide the highest incidence of TBM occurs in the 25-34 year (34%) and 35-44 year (29%) age groups. Mortality due to TBM is also highest in these age groups 33% in 25-34 year age group and 31% in 35-44 year age group. Hence, a separate meta-analysis is needed in adults for the diagnostic accuracy of CSF-ADA in TBM. So, our main objective in this systematic review will be to incorporate those newer studies in adults with the studies in adult population in all the previous meta-analyses to get the final pooled sensitivity and specificity data for the diagnostic accuracy of CSF-ADA in adults suffering from tuberculous meningitis.

## Review

Material and methods

This systematic review was registered in the International Prospective Register of Systematic Reviews (PROSPERO 2022, #CRD42022336559) and is being reported as per the guidelines of the Preferred Reporting Items for Systematic Reviews and Meta-Analysis (PRISMA) [8].

PICO Criteria

We defined the research question by employing the PICO device. Patients (P) were TBM cases, defined as per the definition given in the individual study included in this meta-analysis. Index test (I) was the CSF-ADA and the same cut-off value for the CSF-ADA was used as mentioned in the studies. Comparison (C) was done between the TBM and non-TBM cases as defined by the individual study included in the present meta-analysis. Outcome (O) was diagnostic efficiency, as procured by pooled sensitivity and specificity.

Eligibility Criteria

We included only those observational or cross-sectional and case-control studies from January 1980 to June 2022 that fulfill the following inclusion criteria: (1) reporting sufficient data to determine pooled sensitivity and pooled specificity for the diagnostic accuracy of CSF-ADA in TBM, (2) studies reporting CSF-ADA as a biomarker to differentiate between TBM and non-TBM cases, (3) reporting data in the English language, (4) conducted in age groups more than 18 years of age, (5) published as full-text article, and (6) published as original article. Studies were excluded if they reported as follows: (1) editorial, (2) letter to editor, (3) studies with insufficient data, (4) abstract, (5) case report, (6) pre-print, (7) conference proceeding, and (8) patients less than 18 years of age.

Information Sources

We searched electronic search engines and databases like PubMed, Google Scholar, and Cochrane Library to obtain the relevant articles or studies published until June 2022. In addition, references from eligible studies were also sought for the relevant articles.

Search Strategy

Keywords for the search were CSF OR cerebrospinal fluid AND ADA OR adenosine deaminase AND *Mycobacterium tuberculosis *OR tuberculous AND meningitis AND sensitivity. The filter to search is restricted to human applied.

Selection Process

Two independent reviewers (MKP and AK) searched and selected the published literature and retrieved the desired data from the studies fulfilling the inclusion criteria. Any discrepancy or disagreement was resolved through mutual consensus and discussion.

Data Collection Process

Following data were retrieved from each eligible study by two independent reviewers: first author name, year of publication, sample type (prospective or retrospective), number of patients of TBM and non-TBM, ethnicity of patient (divided into Asian and Caucasian), country of origin, ADA measurement assay method, reference standard (bacteriology with culture/PCR and radio imaging), cut-off value of CSF-ADA, etiology of control group (bacterial, viral, etc.), AUC. HIV position (positive or negative), CSF-ADA mean, mean age, sex, sensitivity, specificity, true positive, false positive, true negative, and false negative data. Any discrepancy was resolved by the mutual consensus of the reviewers. If any information was not available in the study then, the term NA (not available) was used.

Risk of Bias and Applicability

The Quality Assessment of Diagnostic Accuracy Studies-2 (QUADAS-2; Bristol, England: University of Bristol) tool was used to evaluate the scholarly quality of the incorporated studies [9]. It is composed of four key parameters namely patient selection, index test, reference standard, and flow and timing. Each parameter was graded as low, high, or unclear and the first three parameters were further assessed for the applicability concern.

Statistical Analysis

For the diagnostic accuracy, pooled sensitivity and specificity, diagnostic odds ratio, and area under the curve (AUC) with 95% confidence interval were computed to get the effect size by employing a random effect model. AUC was computed from the summary receiver operating characteristic (SROCs) curves to know the discriminatory accuracy of CSF-ADA to diagnose TBM in adults. Chi-square test and I^2^ statistics were used to look for the heterogeneity between different studies. We contemplated the notable p-value of less than 0.05. To ascertain the clinical usefulness of the diagnostic test with positive and negative likelihood ratios, the Fagan plot was utilized.

To seek the cause of heterogeneity, meta-regression and subgroup analysis were executed. In the meta-regression analysis, we considered eight moderator variables as follows: (1) data collection (categorical variable {prospective/retrospective}), (2) reference standard (categorical variable {with imaging/without imaging}), (3) ADA assay method (categorical variable {Guisti/non-Guisti}), (4} ADA cut-off (categorical variable {<10/>10 U/L}), (5) race (categorical variable {Asian/Caucasian}), (6) mean age (continuous variable, in years), (7) sex (categorical variable {male/female}), and (8) sample size (categorical variable {<80/>80}). The significant variables (p<0.05) following meta-regression were considered for subgroup analysis. Publication bias was assessed by using Deek’s funnel plot asymmetry test. To ascertain the quality of evidence for CSF-ADA as a diagnostic marker for TBM the Grading of Recommendations, Assessment, Development, and Evaluation (GRADE) analysis was used. STATA version 13 (College Station, TX: StataCorp LLC) was used for the analysis. Review Manager version 5.4 (Copenhagen, Denmark: the Nordic Cochrane Centre, The Cochrane Collaboration) was used for the risk of bias assessment.

Result

Search Result

**Figure 1:**
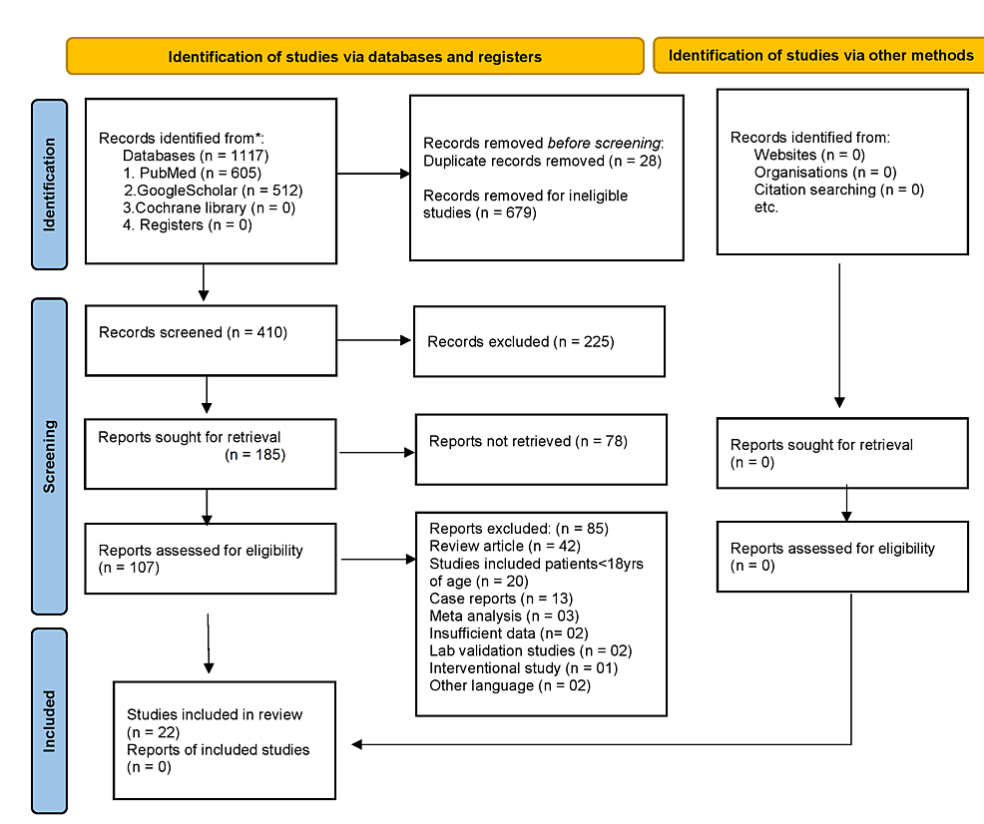
PRISMA flow diagram representing selection and inclusion of different studies. PRISMA: Preferred Reporting Items for Systematic Reviews and Meta-Analyses

A sum of 1117 studies was recognized by electronic databases and search engines (605 from PubMed and 512 from Google Scholar). Most of these were eliminated by evaluating title and abstract. A total of 185 studies remained for full-text review and drawing out data. Eventually, 163 articles were pulled out because of various reasons as mentioned in the PRISMA 2020 flow diagram leaving 22 studies for final analysis (Figure 1).

Characteristics of Studies

Altogether 1927 was the number of study subjects comprising these 22 studies. Of these 22 studies, 20 had prospective data while two had retrospective data. The total number of study subjects who participated in these studies varied from 24 to 190. Mean age of incorporated studies varied from 28.82. years to 50.91 years and the percentage of males varied from 48.35 to 75. Tables 1, 2 dispense the full description of all incorporated studies.

**Table 1:** Characteristics of the studies. ADA: adenosine deaminase; HIV: human immunodeficiency virus; AUC: area under the curve; TBM: tuberculous meningitis; ntb: non-tuberculous; NA: not available; ND: neurological disorders; a: aseptic; b: bacterial; v: viral; f: fungal; p: parasitic Others: other non-infectious conditions leading to meningitis-like symptoms (e.g., multiple sclerosis, Guillain-Barre syndrome, connective tissue disorders, metastatic cancers, etc.).

Author	Year	ADA assay method	Cut-off (U/L)	Etiology of control group	HIV Position	AUC	ADA mean TBM (U/L)	ADA mean non-TBM (U/L)	Race	Country	Mean age (years)	Sex (%) male
Kashyap et al. [10]	2006	Guisti	11.39	b+v	NA	NA	14.31±3.87	9.21±2.14	Asian	India	NA	NA
Kashyap et al. [11]	2007	Guisti (Berthlot reaction)	5	b+ND	NA	NA	15.35±3.46	9.25±2.14	Asian	India	NA	NA
Gautam et al. [12]	2007	Guisti (Berthlot mod)	6.97	NA	NA	NA	13.69±6.49	6.51±2.41	Asian	India	28.82	NA
Bandyopadhyay et al. [13]	2008	Guisti (Berthlot reaction)	10	NA	NA	NA	NA	NA	Asian	India	NA	NA
Rana et al. [14]	2010	Guisti	10	p	Negative	0.992	26.1±19	5.7±3.9	Asian	India	31.3	65.52
Belagavi et al. [15]	2011	Guisti	10	b+v	Positive (2)	NA	14.14±7.44	9.80±13.6	Asian	India	NA	NA
Karsen et al. [16]	2011	Colorimetry	11	b+a	NA	0.965	28.34±14.83	8.71±5.83	Asian	Turkey	32.26	48.35
Nepal et al. [17]	2012	Guisti	8.83	NA	NA	1	11.16±2.03	5.35±1.89	Asian	Nepal	38	NA
Solari et al. [18]	2013	NA	6	v+f+b+p+ND	Positive (38 TBM/34 NTB)	0.82	NA	NA	Caucasian	Peru	NA	70.3
Sharif and Vidya [19]	2014	ADA-MTB kit	10	b+a	NA	NA	16.32±0.71	8.89±1.03	Asian	India	NA	NA
Shadia et al. [20]	2015	ADA assay kit (diazyme)	10	b+others	NA	NA	17.9±2.7	5.3±4.1	Asian	Bangladesh	NA	NA
Parra-Ruiz et al. [21]	2015	Kinetic spectrophotometry	11.5	b+v+others	Positive (2 TBM/13 NTB)	0.84	16.6 median	9.2 b/8.3 v/ 6.3 a median	Caucasian	Spain	NA	55.26
Krishnaswamy et al. [22]	2016	Guisti (reagent-Kaplan)	8	b+others	NA	NA	12.4±6.4	3.5±2.1	Asian	India	NA	NA
Kothari et al. [23]	2017	Colorimetry assay kit	10	NA	NA	NA	11.81	1.65	Asian	India	NA	70.58
Reddy et al. [24]	2017	NA	10	b+a	Negative	NA	13.68	5.76b/8a	Asian	India	31.04 TBM	NA
Raviraj et al. [25]	2017	ADA assay kit (Diazyme)	6.65	NA	NA	NA	10.97±4.43	5.09±1.53	Asian	India	45.06	58.82
Habib et al. [26]	2018	Guisti	10	NA	NA	0.71	NA	NA	Asian	Pakistan	47.09	75
Pathak et al. [27]	2018	NA	NA	b+v	NA	0.92	14.37±6.38	4.02±1.98	Asian	India	NA	NA
Mondal et al. [28]	2018	NA	10	b+v	NA	0.94	31.16	2.03 b/2.46 v	Asian	India	NA	58
Chan et al. [29]	2020	AU 681 chemist analyzer	5.1	a+v+b+f+others	Positive (1 TBM/7 NTB)	0.91	8.6±2.1	2.8±5.91	Asian	Hongkong	NA	56.86
Nand et al. [30]	2020	NA	6	b+v+f+others	NA	NA	10.7±20.24	5.8 b/4.7 v/5 f/4.2 others	Asian	India	50.91	61.97
Anil et al. [31]	2021	NA	6.05	NA	NA	0.92	17.98±8.51	4.62±2.72	Asian	India	NA	67.24

**Table 2:** Characteristics of studies. B: bacteriology; CD: clinical diagnosis; CECT: contrast-enhanced computed tomography; MRI: magnetic resonance imaging; PCR: polymerase chain reaction; CT: computed tomography; ZN: Ziehl-Neelsen; Biochem: biochemical tests; TBM: tuberculous meningitis; TP: true positive; FP: false positive; TN: true negative; FN: false negative; Gm: Gram

Author	Year	TBM	Non-TBM	Sensitivity	Specificity	TP	FN	TN	FP	Data collection	Reference standard
Kashyap et al. [10]	2006	117	60	82	83	96	21	50	10	Prospective	B+CD
Kashyap et al. [11]	2007	66	87	83	86	55	11	75	12	NA	B+CD
Gautam et al. [12]	2007	20	10	70	85	14	6	9	2	NA	B+CD
Bandyopadhyay et al. [13]	2008	44	36	47.7	69.4	21	23	25	11	NA	B+CD+X-ray+Mantoux test
Rana et al. [14]	2010	54	4	92.5	50	50	4	2	2	Prospective	B+CD+CECT+MRI
Belagavi et al. [15]	2011	24	26	73.9	92.6	18	6	24	2	Prospective	B+CD
Karsen et al. [16]	2011	24	67	92	90	22	2	60	7	Prospective	B+CD+MRI
Nepal et al. [17]	2012	8	16	100	93.8	8	0	15	1	Prospective	B+CD+X-ray
Solari et al. [18]	2013	59	96	55.9	95	33	26	91	5	Prospective	B+CD
Sharif and Vidya [19]	2014	25	31	94.73	90.47	24	1	28	3	Prospective	B+CD
Shadia et al. [20]	2015	27	10	77.77	80	21	6	8	2	Prospective	B+CD+PCR+X-ray
Parra-Ruiz et al. [21]	2015	11	179	91	77.7	10	1	139	40	Retrospective	B+CD
Krishnaswamy et al. [22]	2016	25	60	95	92	24	1	55	5	Prospective	B+CD
Kothari et al. [23]	2017	17	69	64.7	97	11	6	67	2	Prospective	B+CD
Reddy et al. [24]	2017	25	50	100	92	25	0	46	4	Prospective	B+CD+CT
Raviraj et al. [25]	2017	34	51	85.3	84.3	29	5	43	8	Prospective	B+CD+PCR+CECT+MRI
Habib et al. [26]	2018	114	22	84.21	95.45	96	18	21	1	Prospective	B+CD+PCR+CT
Pathak et al. [27]	2018	23	27	82.61	100	19	4	27	0	Prospective	B+CD
Mondal et al. [28]	2018	19	31	89.47	96.77	17	2	30	1	Prospective	Gm stain+ZN stain+biochem+CT
Chan et al. [29]	2020	8	43	100	91	8	0	39	4	Retrospective	B+CD
Nand et al. [30]	2020	30	120	56.6	97.5	17	13	117	3	Prospective	B+CD+CT+MRI
Anil et al. [31]	2021	12	46	91.7	63	11	1	29	17	Prospective	B+CD

Methodological Quality

**Figure 2:**
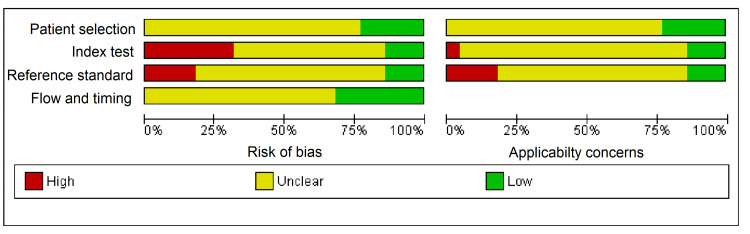
Risk of bias and applicability concerns graph: review author's judgments about each domain presented as percentages across included studies.

**Figure 3:**
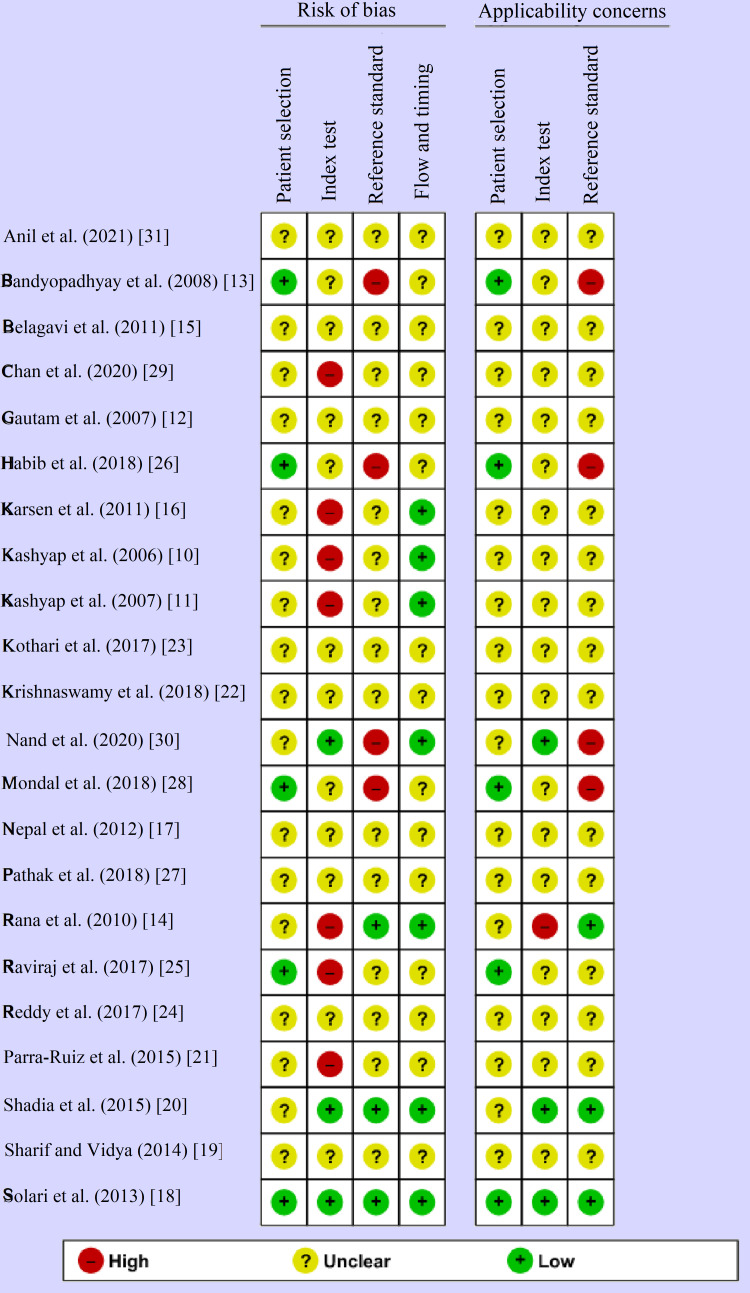
Risk of bias and applicability concerns summary: review author's judgment about each domain for each included study.

About 75% of studies had not reported the exact details of the patient selection method. More than 30% studies were biased about the index test as it was unclear whether the researcher was blinded about the interpretation of the index test or due to lack of a pre-specified threshold used for the index test. More than 20% studies were biased about the reference standard as they had not reported the correct reference test to classify the target condition. The full description of the evaluation by QUADAS-2 device is given in Figures 2, 3.

Diagnostic Performance of CSF-ADA for TBM

**Figure 4:**
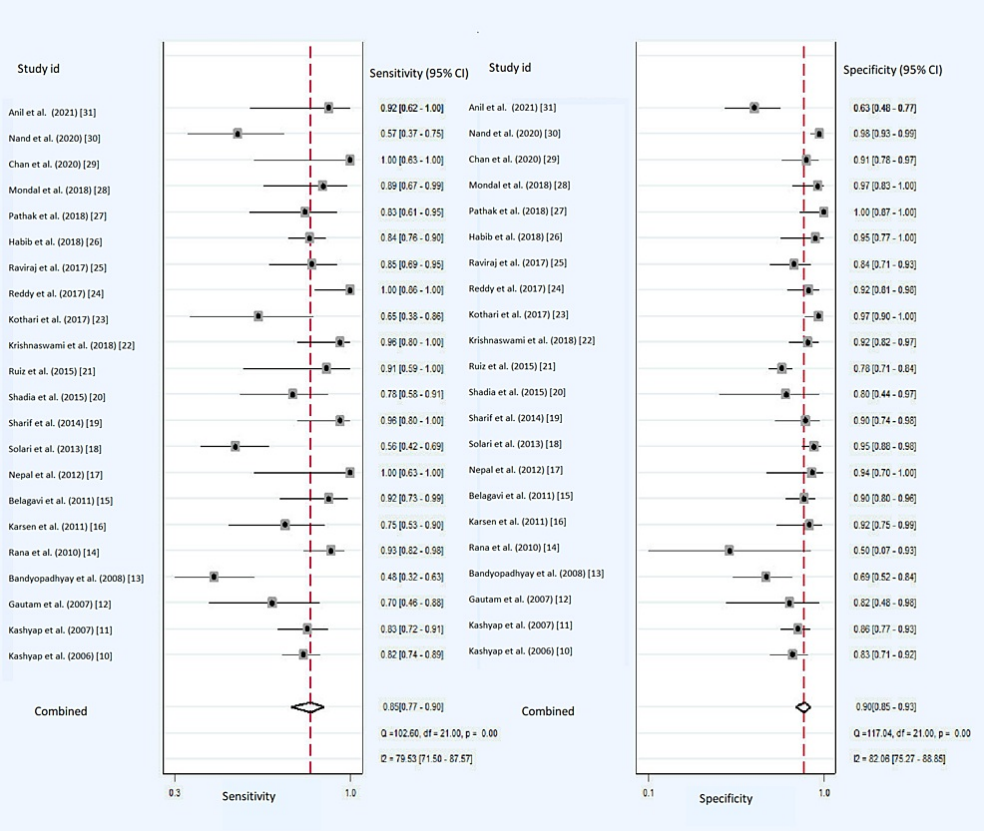
Forest plot of pooled sensitivity and pooled specificity of CSF-ADA in the diagnosis of TBM. CSF: cerebrospinal fluid; ADA: adenosine deaminase; TBM: tuberculous meningitis

**Figure 5:**
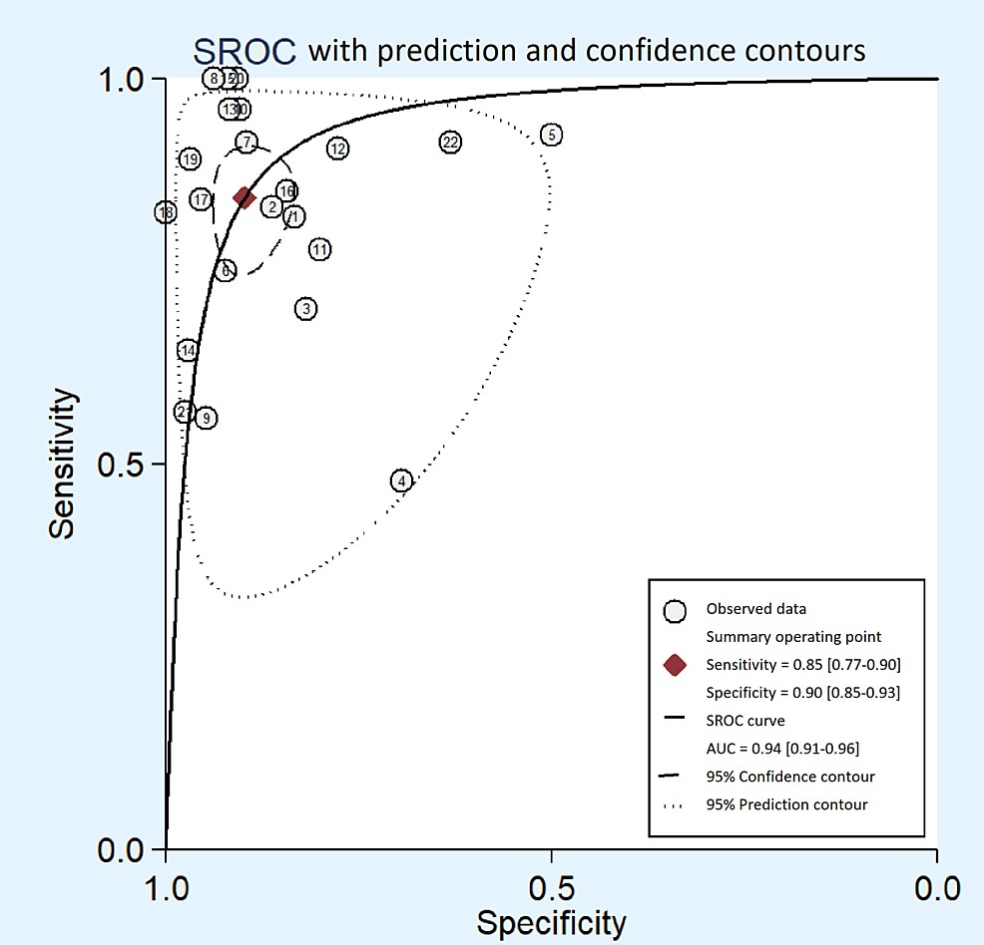
Summary ROC curve with prediction and confidence contours showing the discriminatory power of CSF-ADA for diagnosis of TBM. ROC: receiver operating characteristic; CSF: cerebrospinal fluid; ADA: adenosine deaminase; TBM: tuberculous meningitis

**Figure 6:**
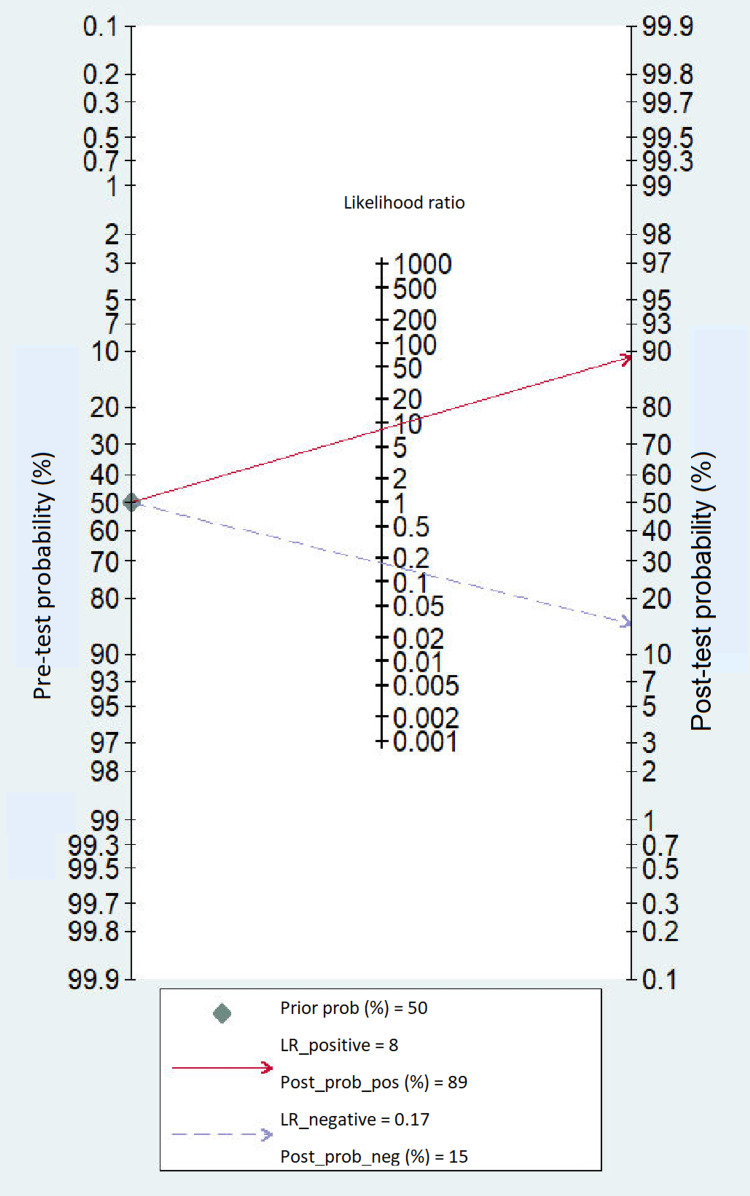
Fagan nomogram of CSF-ADA. Nomogram analysis showing pre-test and post-test probability of CSF-ADA in the diagnosis of TBM. CSF: cerebrospinal fluid; ADA: adenosine deaminase; TBM: tuberculous meningitis

The sensitivities and specificities of the individual studies are shown in Table 2, for the diagnosis of TBM. Encouraging result was observed for the diagnostic accuracy of CSF-ADA to diagnose TBM in adults with summary estimates of sensitivity as 0.85 (95% CI: 0.77-0.90), specificity as 0.90 (95% CI: 0.85-0.93) and diagnostic odds ratio of 48 (95% CI: 26-86) (Figure 4). The accuracy to discriminate was also promising (SROC curves 0.94 {95% CI: 0.91-0.96}) to differentiate TBM from non-TBM patients (Figure 5). Based on our clinical experience, if we consider the pre-test probability of 50% for diagnosing TBM with the CSF-ADA test, it will increase the post-test probability up to 89% with positive likelihood ratio (PLR) of 8 (95%: CI 5.7-11.9) and negative likelihood ratio (NLR) of 0.17 (95% CI: 0.12-0.26) and post-test probability of 15% as shown in Fagan plot (Figure 6).

Publication Bias

**Figure 7:**
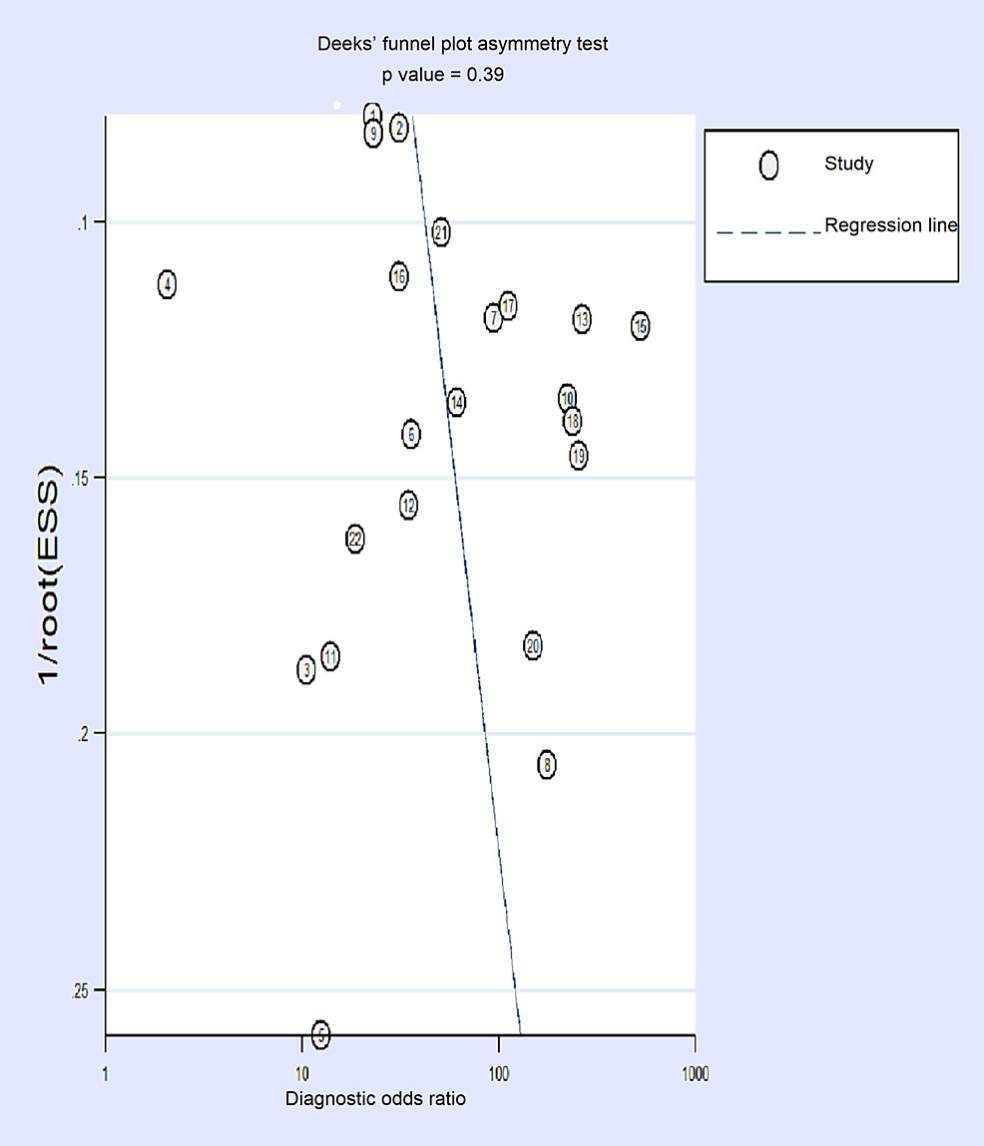
Deek's funnel plot for assessing the risk of publication bias.

The risk of publication bias was evaluated by composing Deek’s funnel plot which affirms the absence of any notable publication bias (p=0.39) (Figure 7).

Meta-Regression and Subgroup Analysis

**Figure 8:**
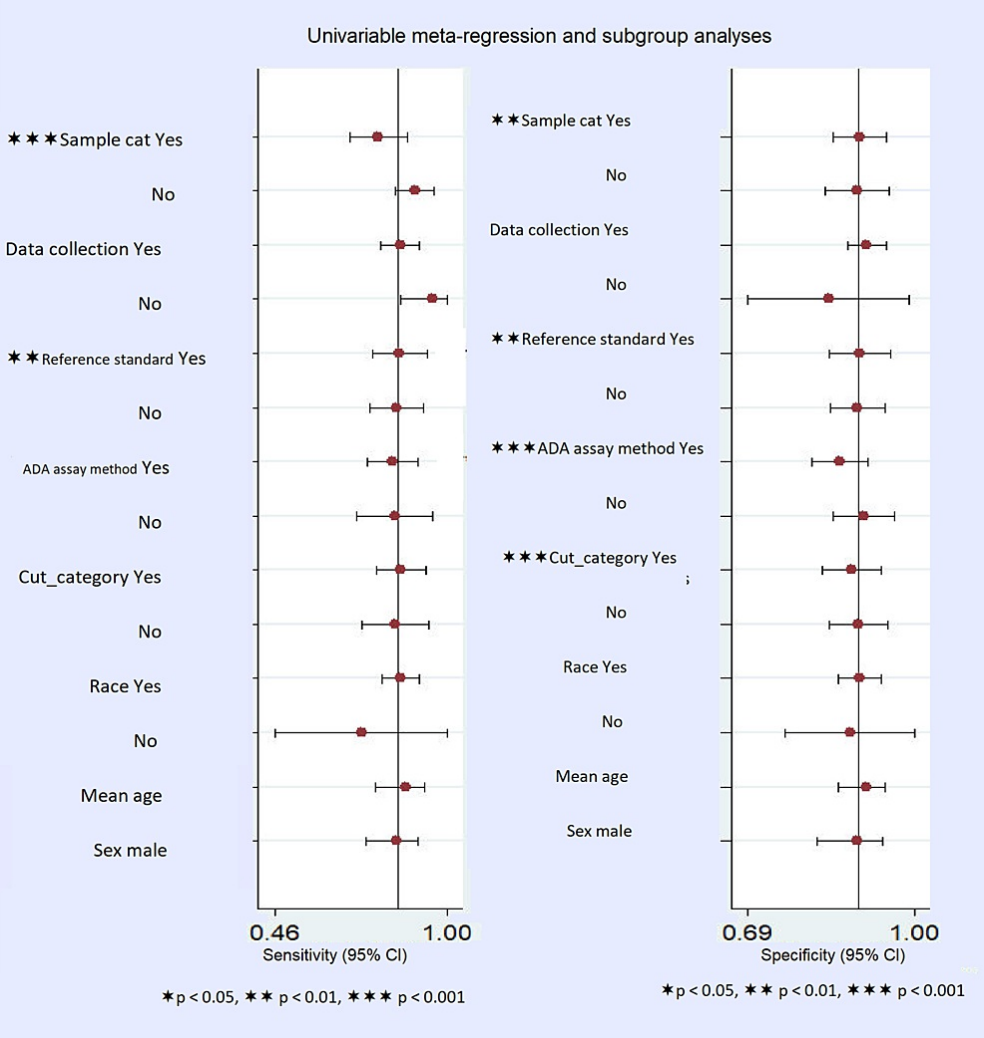
Meta-regression analysis showing the source of heterogeneity might be due to the sample size, reference standard, ADA assay method, and CSF-ADA cut-off value in the study. CSF: cerebrospinal fluid; ADA: adenosine deaminase

To look for the variables which could disclose the origin of heterogeneity meta-regression analysis was carried out. The analysis suggested that sample size (<80 and >80), reference test, CSF-ADA cut-off, and type of ADA assay methods might be sources of the heterogeneity (Figure 8). Therefore, subgroup analysis was executed (Table 3). In this analysis, regarding the ADA assay method, nine studies have used the older Guisti method while seven studies have used non-Guisti method that suggested that non-Guisti method had a higher diagnostic odds ratio (dOR=51) with heterogeneity I^2^=74.5% for specificity and I^2^=51.1% for sensitivity, while comparatively lower diagnostic odds ratio in Guisti method (dOR=32) with I^2^=60% in specificity and I^2^=83.4% in the sensitivity for diagnosing tuberculous meningitis using CSF-ADA (Table 3).

**Table 3:** Subgroup analysis for diagnostic accuracy of CSF-ADA in tuberculous meningitis. CSF: cerebrospinal fluid; ADA: adenosine deaminase; PLR: positive likelihood ratio; NLR: negative likelihood ratio; DOR: diagnostic odds ratio; AUC: area under the curve

Subgroup		Number of studies	Sensitivity (95% CI)	Specificity (95% CI)	Heterogeneity (95% CI)		PLR	NLR	DOR	AUC (95% CI)
					Sensitivity	Specificity				
CSF-ADA cut-off	<10	9	0.82 (0.69-0.91)	0.90 (0.83-0.94)	78.04 (64.05-92.03)	84.22 (75.00-93.44)	7.9	0.2	40	0.93 (0.90-0.95)
	>10	12	0.85 (0.76-0.91)	0.88 (0.82-0.93)	82.46 (73.41-91.50)	78.69 (67.11-90.26)	7.4	0.17	45	0.93 (0.91-0.95)
Sample size	<80	11	0.90 (0.81-0.94)	0.90 (0.81-0.94)	57.44 (28.89-85.99)	76.68 (63.11-90.26)	8.5	0.12	73	0.95 (0.93-0.97)
	>80	11	0.79 (0.68-0.86)	0.90 (0.84-0.94)	83.47 (74.69-92.25)	84.11 (75.76-92.46)	7.8	0.24	33	0.92 (0.89-0.94)
Reference standard	Without radio imaging	12	0.83 (0.74-0.89)	0.89 (0.83-0.93)	71.15 (54.21-88.09)	82.97 (74.26-91.68)	7.8	0.19	41	0.93 (0.90-0.95)
	With radio imaging	10	0.86 (0.73-0.93)	0.90 (0.83-0.95)	86.64 (79.59-93.68)	80.08 (68.37-91.78	8.8	0.16	57	0.94 (0.92-0.96)
ADA assay method	Non-Guisti	7	0.87 (0.77-0.93)	0.89 (0.82-0.93)	51.10 (9.21-92.98)	74.58 (56.37-93.78)	7.6	0.15	51	0.94 (0.92-0.96)
	Guisti	9	0.83 (0.73-0.90)	0.86 (0.80-0.91)	83.46 (73.68-93.24)	60.14 (30.96-89.32)	6.1	0.19	32	0.91 (0.89-0.94)

Subgroup analysis based on sample size (>80 and <80) suggested that sample size <80 (11 studies) had diagnostic odds ratio of 73 (I^2^=57.44% for sensitivity and I^2^=76.68% for specificity). On the other hand, the diagnostic odds ratio of 33 with heterogeneity I^2^=83.4% for sensitivity and I^2^=84.1% for specificity. The variation according to the differences in the reference method used and cut-off value for CSF-ADA was found to be minimal.

GRADE Analysis

The certainty of evidence shown by our GRADE analysis was found to be of very low quality both for the sensitivity and specificity of cerebrospinal fluid adenosine deaminase to diagnose tuberculous meningitis (Table 4).

**Table 4:** Grading of Recommendations, Assessment, Development, and Evaluation (GRADE) analysis. Sensitivity: 0.85 (95% CI: 0.77 to 0.90); specificity: 0.90 (95% CI: 0.85 to 0.93); prevalence: 10%, 30%, 50%. CoE: certainty of evidence

Outcome	No. of studies and no. of patients	Study design	Factors that may decrease certainty of evidence					Effect per 100 patients tested			Test accuracy CoE
			Risk of bias	Indirectness	Inconsistency	Imprecision	Publication bias	Pre-test probability of 10%	Pre-test probability of 30%	Pre-test probability of 50%	
True positives (patients with [target condition} TBM)	22 studies 1927 patients	Cohort and case-control type studies	Very serious	Not serious	Very serious	Not serious	All plausible residual confounding would reduce the demonstrated effect	9 (8-9)	26 (23-27)	43 (39-45)	⨁◯◯◯ Very low (certainty of evidence)
False negatives (patients incorrectly classified as not having {target condition} TBM)	22 studies 1927 patients	Cohort and case-control type studies	Very serious	Not serious	Very serious	Not serious	All plausible residual confounding would reduce the demonstrated effect	1 (1-2)	4 (3-7)	7 (5-11)	
True negatives (patients without {target condition} TBM)	22 studies 1927 patients	Cohort and case-control type studies	Very serious	Not serious	Very serious	Not serious	All plausible residual confounding would reduce the demonstrated effect	81 (77-84)	63 (60-65)	45 (43-47)	⨁◯◯◯ Very low (certainty of evidence)
False positives (patients incorrectly classified as having {target condition} TBM)	22 studies 1927 patients	Cohort and case-control type studies	Very serious	Not serious	Very serious	Not serious	All plausible residual confounding would reduce the demonstrated effect	9 (6-13)	7 (5-10)	5 (3-7)	

Discussion

Examination of CSF-ADA is the most common immunodiagnostic method for TBM. It is a rapid, cheap, and easily accessible method to diagnose TBM [5]. In the past, many studies have been accomplished to secure the anticipated accuracy of CSF-ADA to diagnose TBM, although their sensitivity and specificity differ much.

Xu et al., Tuon et al., and Pormohammad et al. previously published meta-analyses to explore this research question with a smaller number of studies [6,32,33]. Xu et al. and Tuon et al. published meta-analyses only with 10 and 13 studies, respectively, and Pormohammad et al. with 20 studies (Table 5). They all used pooled sensitivity, pooled specificity, summary area under the curve, and diagnostic odds ratio to report their meta-analysis. However, for methodological quality assessment, QUADAS-2 was used only by Pormohammad et al. [6]. For the analysis of publication bias, Egger’s test was used by Xu et al. and Tuon et al., while Deek’s funnel test was used by Pormohammad et al. [6,32,33]. Meta-regression was only done by Xu et al., while sensitivity analysis was done by Pormohammad et al. [6,32]. Sub-group analysis and grade analysis have been done which were not done in previous studies which have improved this study (Table 5).

**Table 5:** Comparison of the present meta-analysis and previous meta-analyses. QUADAS: Quality Assessment of Diagnostic Accuracy Studies; GRADE: Grading of Recommendations, Assessment, Development, and Evaluation

Criteria		Xu et al. (2010) [32]	Tuon et al. (2010) [33]	Pormohammad et al. (2017) [6]	Present meta-analysis
Number of studies		10	13	20	22
Number of participants		TBM: 375, non-TBM: 989	TBM: 380, non-TBM: NA	TBM: 741, non-TBM: 1169	TBM: 786, non-TBM: 1141
Age group		Adult+children	Adult+children	Adult+children	Adults only
Recommended guidelines for reporting meta-analysis	Pooled sensitivity	Yes	Yes	Yes	Yes
	Pooled specificity	Yes	Yes	Yes	Yes
	Summary area under the curve	Yes	Yes	Yes	Yes
	Diagnostic odds ratio	Yes	Yes	Yes	Yes
	Methodological quality (QUADAS-2)	No	No	Yes	Yes
	Publication bias	Yes (Egger’s test, funnel plot)	Yes (Egger’s test)	Yes (Deek’s funnel test)	Yes (Deek’s funnel test)
	Meta-regression	Yes	No	No	Yes
	Sub-group analysis	No	No	No	Yes
	GRADE analysis	No	No	No	Yes
Analysis used		Pooled sensitivity, pooled specificity, summary area under the curve, diagnostic odds ratio, meta-regression	Pooled sensitivity, pooled specificity, summary area under the curve, diagnostic odds ratio	Pooled sensitivity, pooled specificity, summary area under the curve, diagnostic odds ratio, sensitivity analysis	Pooled sensitivity, pooled specificity, summary area under the curve, diagnostic odds ratio, meta-regression, subgroup analysis

We have reported our meta-analysis with all these details of pooled sensitivity, pooled specificity, summary area under the curve, diagnostic odds ratio, QUADAS-2, publication bias, meta-regression, sub-group analysis, and GRADE analysis (Table 5).

These previously done meta-analyses have included patients of all age groups. Till now, there is no separate meta-analysis investigating this research question in adults. Considering the following facts: (1) the higher number and mortality of TBM cases are in the adult population [2]. (2) Increased chances of variable CSF examination result due to higher incidence of traumatic lumbar puncture in neonates, infants, and children [7]. (3) Several new studies have been published after the publication of these meta-analyses. There is a dire need to explore this research question in adults.

Our meta-analysis has explored this research question only in adults over 18 years of age by incorporating the studies from the previously published meta-analyses along with the newer studies, having this age group of patients from January 1980 to June 2022.

Our meta-analysis includes total of 22 studies exhibiting remarkable diagnostic accuracy of CSF-ADA for detecting TBM cases with a sensitivity of 0.85 (95% CI: 0.77-0.90), specificity of 0.90 (95% CI: 0.85-0.93), area under curve (AUC) 0.94 (95% CI: 0.91-0.96), and diagnostic odds ratio 48 (95% CI: 26-86) thus, building it a promising diagnostic test for TBM in adult patients.

Regarding our subgroup analysis, there was not any remarkable distinctness in the inequitable potential between studies with a cut-off value of <10 U/L and studies with a cut-off value of >10 U/L for diagnosing TBM. This finding is compatible with an earlier meta-analysis Tuon et al., reinforcing the justifiability of taking into consideration the cut-off value of CSF-ADA 10 U/L for the diagnosis of TBM [33]. Increased number of false-negative values are encountered when we choose higher cut-off values hence lower cut-off value is advised. Our statistics stipulate that a cut-off value of 10 U/L attains the same diagnostic efficiency as a higher cut-off value. Hence, we may consider 10 U/L as the ideal cut-off value for CSF-ADA to diagnose TBM.

In our meta-analysis, the methods used for ADA assay in the included studies were categorized into Guisti and non-Guisti methods. Guisti method is an older established method while the various non-Guisti methods (kit method, AU681 chemist analyzer, and kinetic spectrophotometry) are newer and easy to use. Non-Guisti methods had a higher diagnostic odds ratio (dOR=51) than the Guisti methods (dOR=32) signifying that the assessment of CSF-ADA by non-Guisti methods had a higher diagnostic accuracy than the Guisti methods to diagnose TBM.

Subgroup analysis further revealed that the CSF-ADA had a higher diagnostic accuracy to diagnose TBM for the sample size <80 (dOR=73) than the sample size >80 (dOR=33) which is contrary to the belief that higher sample size gives better results. For this, we need individual patient data analysis to reach an optimal conclusion.

Strength

The solidity and validity are the major strength of this study which are achieved by adhering to the protocol for the strategy and coverage of meta-analysis for diagnostic test accuracy. This is the first meta-analysis in adults analyzing the diagnostic accuracy of CSF-ADA in cases of tuberculous meningitis containing more than 50% of the included studies which have been published recently. Assessment of the quality of methodology of the included studies done by utilizing a suitable tool (QUADAS-2) [9]. We employed meta-regression and subgroup analysis to look for the potential source of heterogeneity. Further, grade analysis has been done in our meta-analysis to look for the certainty of evidence for the pooled sensitivity and specificity.

Limitations

Statistically more reliable result is derived from the individual patient data meta-analysis. Most of the studies were from Southeast Asia which may be due to the higher incidence of TBM cases in this region [2]. Included studies were only in the English language hence language bias could be there in study selection. Our meta-analysis could not analyze the correlation between the TBM and CSF-ADA. The major limitation of this meta-analysis is the lack of a predefined cut-off of CSF-ADA to diagnose TBM, different cut-off has been taken by different studies ranging from as low as 5 to as high as 11.5.

## Conclusions

After going through the present meta-analysis, we came to the conclusion that CSF-ADA is a promising diagnostic test with high specificity and admissible sensitivity for the diagnosis of tuberculous meningitis in adults, however, with very low level of certainty of evidence. A total of 10 U/L may be taken as an ideal cut-off value for cerebrospinal fluid adenosine deaminase to diagnose tuberculous meningitis in adults in areas having high prevalence of tuberculosis. However, to have a common consensus regarding a definite cut-off for CSF-ADA all over the world, we need more studies from other parts of the world apart from the Southeast Asian region. Thus, we wrap up this part with the expectation that in the coming times we will have an absolute diagnostic test to diagnose tuberculous meningitis at the earliest to save more life from this serious complication of tuberculosis.

## References

[1] (2022). Tuberculosis. https://www.who.int/news-room/fact-sheets/detail/tuberculosis.

[2] Dodd PJ, Osman M, Cresswell FV (2021). The global burden of tuberculous meningitis in adults: a modelling study. PLOS Glob Public Health.

[3] Bartzatt R (2011). Tuberculosis infections of the central nervous system. Cent Nerv Syst Agents Med Chem.

[4] Bahr NC, Boulware DR (2014). Methods of rapid diagnosis for the etiology of meningitis in adults. Biomark Med.

[5] Gupta BK, Bharat A, Debapriya B, Baruah H (2010). Adenosine deaminase levels in CSF of tuberculous meningitis patients. J Clin Med Res.

[6] Pormohammad A, Riahi SM, Nasiri MJ, Fallah F, Aghazadeh M, Doustdar F, Pouriran R (2017). Diagnostic test accuracy of adenosine deaminase for tuberculous meningitis: a systematic review and meta-analysis. J Infect.

[7] Lee TJ, Aronson PL (2018). To spinal tap or not to spinal tap, that is the question. Hosp Pediatr.

[8] Moher D, Liberati A, Tetzlaff J, Altman DG (2009). Preferred reporting items for systematic reviews and meta-analyses: the PRISMA statement. J Clin Epidemiol.

[9] Whiting PF, Rutjes AW, Westwood ME (2011). QUADAS-2: a revised tool for the quality assessment of diagnostic accuracy studies. Ann Intern Med.

[10] Kashyap RS, Kainthla RP, Mudaliar AV, Purohit HJ, Taori GM, Daginawala HF (2006). Cerebrospinal fluid adenosine deaminase activity: a complimentary tool in the early diagnosis of tuberculous meningitis. Cerebrospinal Fluid Res.

[11] Kashyap RS, Ramteke SP, Deshpande PS, Purohit HJ, Taori GM, Daginawala HF (2007). Comparison of an adenosine deaminase assay with ELISA for the diagnosis of tuberculous meningitis infection. Med Sci Monit.

[12] Gautam N, Aryal M, Bhatta N, Bhattacharya SM, Baral N, Lamsal M (2007). Comparative study of cerebrospinal fluid adenosine deaminase activity in patients with meningitis. Nepal Med Coll J.

[13] Bandyopadhyay D, Gupta S, Banerjee S, Gupta S, Ray D, Bhattacharya S, Bhattacharya B (2008). Adenosine deaminase estimation and multiplex polymerase chain reaction in diagnosis of extra-pulmonary tuberculosis. Int J Tuberc Lung Dis.

[14] Rana SV, Chacko F, Lal V, Arora SK, Parbhakar S, Sharma SK, Singh K (2010). To compare CSF adenosine deaminase levels and CSF-PCR for tuberculous meningitis. Clin Neurol Neurosurg.

[15] Belagavi AC, Shalini M (2011). Cerebrospinal fluid C-reactive protein and adenosine deaminase in meningitis in adults. J Assoc Physicians India.

[16] Karsen H, Koruk ST, Karahocagil MK, Calisir C, Baran FC (2011). Comparative analysis of cerebrospinal fluid adenosine deaminase activity in meningitis. Swiss Med Wkly.

[17] Nepal AK, Gyawali N, Poudel B (2012). Adenosine deaminase in CSF and pleural fluid for diagnosis of tubercular meningitis and pulmonary tuberculosis. Nepal Med Coll J.

[18] Solari L, Soto A, Agapito JC (2013). The validity of cerebrospinal fluid parameters for the diagnosis of tuberculous meningitis. Int J Infect Dis.

[19] Shariff MH and Vidya Pai (2014). Cerebrospinal fluid-adenosine deaminase activity in tuberculous meningitis cases. Int J Pharm Biol Sci.

[20] Shadia K, Kamal SM, Saleh AA, Hossain MN, Gupta RD, Miah RA (2015). Adenosine deaminase assay in different body fluids for the diagnosis of tubercular infection. Am J Biomed Sci.

[21] Parra-Ruiz J, Ramos V, Dueñas C (2015). Rational application of adenosine deaminase activity in cerebrospinal fluid for the diagnosis of tuberculous meningitis. Infection.

[22] Krishnaswamy DR and Priyadharshini K (2016). Diagnostic accuracy of cerebrospinal fluid adenosine deaminase activity in tuberculous meningitis. J Med Sci Clin Res.

[23] Kothari K, Mistry MA, Goswami YS (2017). Utility of ADA (adenosine deaminase) enzyme assay in diagnosis of tuberculous meningitis. Indian J Microbiol Res.

[24] Reddy KB, Durbesula AT, Usham G (2017). Study of adenosine deaminase levels in TB meningitis and its comparision with other types of meningitis. Ann Trop Med Public Health.

[25] Raviraj Raviraj, Henry RA, Rao GG (2017). Determination and validation of a lower cut off value of cerebrospinal fluid adenosine deaminase (CSF-ADA) activity in diagnosis of tuberculous meningitis. J Clin Diagn Res.

[26] Habib A, Amin ZA, Raza SH, Aamir S (2018). Diagnostic accuracy of cerebrospinal fluid adenosine deaminase in detecting tuberculous meningitis. Pak J Med Sci.

[27] Pathak JM, Seth S, Thakkar ZK, Iyer CC, Prajapati KV (2018). Cerebrospinal fluid C-reactive protein and adenosine deaminase levels in the differential diagnosis of meningitis in adults. Int J Contemp Med Res.

[28] Mondal SS, Sen I, Chandra A, Das M (2018). The use of cerebrospinal fluid C-reactive protein and adenosine deaminase as diagnostic markers in differential diagnosis of meningitis. J Med Sci Clin Res.

[29] Chan TC, Chen SP, Mak CM, Ching CK, Luk KS, Tsang YM, Leung DC (2020). Determination of cerebrospinal fluid adenosine deaminase activity cut-off for the diagnosis of tuberculous meningitis in Hong Kong. J Clin Pathol.

[30] Nand L, Chauhan PS, Sharma S, Shandil R (2020). Spectrum of cerebrospinal fluid analysis and nervous system diseases in hospitalised patients: a hospital based prospective study from a hilly state of Himachal Pradesh in North India. Int J Adv Med.

[31] Anil AA, Singh M, Ramakrishnan SR, Roy NR (2021). Cerebrospinal fluid adenosine deaminase and lactate levels as a rapid diagnostic marker for tuberculous meningitis among adults. Ann Romanian Soc Cell Biol.

[32] Xu HB, Jiang RH, Li L, Sha W, Xiao HP (2010). Diagnostic value of adenosine deaminase in cerebrospinal fluid for tuberculous meningitis: a meta-analysis. Int J Tuberc Lung Dis.

[33] Tuon FF, Higashino HR, Lopes MI, Litvoc MN, Atomiya AN, Antonangelo L, Leite OM (2010). Adenosine deaminase and tuberculous meningitis - a systematic review with meta-analysis. Scand J Infect Dis.

